# Mycoloop: chytrids in aquatic food webs

**DOI:** 10.3389/fmicb.2014.00166

**Published:** 2014-04-22

**Authors:** Maiko Kagami, Takeshi Miki, Gaku Takimoto

**Affiliations:** ^1^Faculty of Science, Toho UniversityFunabashi, Japan; ^2^Institute of Oceanography, National Taiwan UniversityTaipei, Taiwan

**Keywords:** parasitic fungi, chytridiomycota, diatom, daphnia, mycoloop, indirect mutualism, stability, trophic transfer

## Abstract

Parasites are ecologically significant in various ecosystems through their role in shaping food web structure, facilitating energy transfer, and controlling disease. Here in this review, we mainly focus on parasitic chytrids, the dominant parasites in aquatic ecosystems, and explain their roles in aquatic food webs, particularly as prey for zooplankton. Chytrids have a free-living zoosporic stage, during which they actively search for new hosts. Zoospores are excellent food for zooplankton in terms of size, shape, and nutritional quality. In the field, densities of chytrids can be high, ranging from 10^1^ to 10^9^ spores L^−1^. When large inedible phytoplankton species are infected by chytrids, nutrients within host cells are transferred to zooplankton via the zoospores of parasitic chytrids. This new pathway, the “mycoloop,” may play an important role in shaping aquatic ecosystems, by altering sinking fluxes or determining system stability. The grazing of zoospores by zooplankton may also suppress outbreaks of parasitic chytrids. A food web model demonstrated that the contribution of the mycoloop to zooplankton production increased with nutrient availability and was also dependent on the stability of the system. Further studies with advanced molecular tools are likely to discover greater chytrid diversity and evidence of additional mycoloops in lakes and oceans.

## Ecological significance of parasites

Parasites are important components of ecological communities (Thomas et al., 2005; Hatcher and Dunn, 2011). They have the potential to regulate host populations, mediate interspecific competition between hosts and other species, maintain genetic polymorphism and biodiversity, and affect community structure. Nevertheless, the effects of parasites and diseases on food webs and ecosystem dynamics have been neglected until recently (Polis and Strong, 1996; Marcogliese and Cone, 1997). New research suggests that parasites have the potential to alter food-web topology, stability, interaction strength and energy flow (Lafferty, 2006; Kuris et al., 2008; Lafferty et al., 2008).

**Figure 1:**
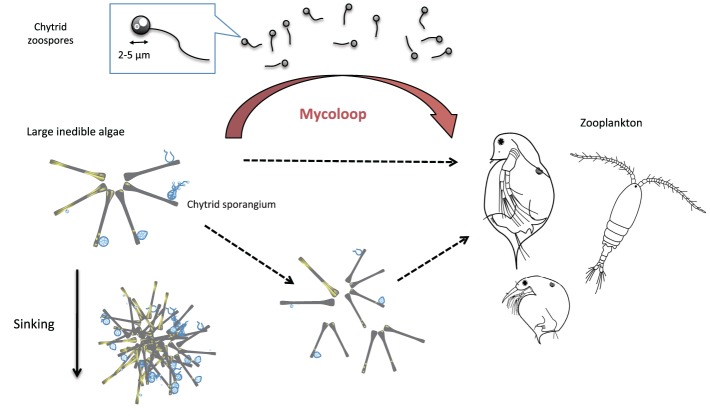
**Diagram of “mycoloop.”** Parasitic chytrids can transfer material from large inedible phytoplankton to zooplankton. Chytrids zoospores are excellent food for zooplankton in terms of size (2–5 μm in diameter), shape, nutritional quality (rich in PUFAs and cholesterols). Large colonies of host phytoplankton may also be fragmented by chytrid infections and become edible to zooplankton. On the other hand, infected host colonies may remain inedible to *Daphnia*, or even become less edible due to the aggregate formation of cells. Those aggregations may sink faster, and affect material cycling in lakes.

Parasites commonly function as prey within ecosystems (Johnson et al., 2010; Thieltges et al., 2013). There are two main ways in which parasites become prey. Predators can either consume the infected hosts of parasites (concomitant predation) or their free-swimming life stage (Johnson et al., 2010). Many aquatic parasites including viruses, chytrids, trematodes, and nematodes, have a free-swimming stage that may be subject to predation (Gonzalez and Suttle, 1993; Kagami et al., 2004; Kuris et al., 2008; Johnson et al., 2010). The Chytridiomycota (chytrids) are one of the dominant groups of parasites in aquatic ecosystems. The free-living zoosporic stage of chytrids actively searches for and infects host cells, extracting nutrients and developing into mature sporangia that release new zoospores (Canter, 1967; Figure 1). There are more than 700 species of chytrids known to infect phytoplankton, zooplankton, fungi, plants, and invertebrate animals (Sparrow, 1960; Gleason et al., 2008). Here in this review, we mainly focus on parasitic chytrids that infect phytoplankton, and explain their roles in aquatic food webs as prey for zooplankton through the “mycoloop” pathway (Kagami et al., 2007a).

## Chytrids zoospores as prey for zooplankton: food quality and quantity, and the mycoloop

Predation of parasites can be beneficial to predators if they can gain energy and nutrition from parasites. Chytrid zoospores are a good food source for zooplankton in terms of size and shape (Kagami et al., 2004). In addition, zoospores are rich in polyunsaturated fatty acids (PUFAs) and cholesterol, which are essential for the growth of crustaceans (Kagami et al., 2007b). Zooplankton, such as cladocerans (*Daphnia*) and copepods, are able to grow by acquiring important supplementary nutrients from a diet of zoospores (Kagami et al., 2007b, 2011). Thus, chytrids may improve zooplankton production and enhance trophic transfer.

Many recent studies suggest that parasite biomass is not negligible, and may in fact be often significantly high (Kuris et al., 2008). The abundance of chytrids in aquatic systems has been found to be much higher than traditionally thought. Because morphological identification of chytrid zoospores is difficult, attempts have been made to use the fluorescent stains to count the density of chytrid zoospores in lakes (Kudoh, 1990). Recently, molecular techniques, such as CARD-FISH (Jobard et al., 2010) and Real-Time qPCR (Lefèvre et al., 2010) have been applied to estimate zoospore abundance in field samples and have detected zoospore concentrations of 10^1^–10^6^ spores L^−1^ (Table 1).

**Table 1:** **Density of zoospores or sporangia of chytrids in lakes**.

**Zoospores (10^3^ L^−1^)**		**Sporangia (10^3^ L^−1^)**		**Methods**	**Chytrid**	**Host**	**Lakes (Trophic status)**	**References**
**min**	**max**	**min**	**max**					
1	360			Count with Nile Red and DAPI	*Rhizophydium, Zygorhizidium*	*Asterionella Formosa*	Lake Suwa, Japan (E)	Kudoh, 1990
89 ± 11	156 ± 51			CARD-FISH (<25 um)	Chytridiales (Rhizophidium, Chytridium^*^)	*Melosira, Anabaena*	Lake Aydat, France (E)	Jobard et al., 2010
52 ± 11	573 ± 68			CARD-FISH (<25 um)	Chytridiales (Rhizophidium, Chytridium^*^)	Various species^a^	Lake Pavin, France (OM)	Jobard et al., 2010
0.04	5			qPCR	Rhizophidiales (parasitic and saprotrophic)	Unknown	Lake Pavin, France (OM)	Lefèvre et al., 2010
0.019	0.454			qPCR	*Badrachochytrium dendrobatidis*	Amphibians	Lakes and ponds, USA	Kirshtein et al., 2007
			1085	Direct count (Utermöhl)	*Zygorhizidium. planktonicum*	*Asterionella Formosa*	Lake Maarsseveen, The Netherlands (OM)	Van Donk and Ringelberg, 1983
			510	Direct count (Utermöhl)	*Zygorhizidium affluens*	*Asterionella Formosa*	Crose Mere, UK	Reynolds, 1973
		1	562	Count with CFW	Zygorhizidium^*^, Chytridium^*^	*Aulacoseira granulata, A. ambigua*	Lake Inba, Japan (E)	Kagami et al., 2012
		0.524	368	Count with CFW	*Rhizophidium, Chytridium, Zygorizihidium*	Various species^b^	Lake Pavin, France (OM)	Rasconi et al., 2012
			31500	Count with CFW	*Rhizophidium, Chytridium, Zygorizihidium*	Various species^c^	Lake Aydat, France (E)	Rasconi et al., 2012
		1	120	Host density × %	*Rhizophydium, Zygorhizidium*	*Asterionella Formosa*	Lake Suwa, Japan (E)	Kudoh and Takahashi, 1990
		0.4	65	Host density × %	Rhizophydium^*^	*Staurastrum dorsidentiferum*	Lake Biwa, Japan (M)	Kagami and Urabe, 2002
			40	Host density × %	*Rhizidium microcystidis*	*Microcystis aeruginosa*	Shearwater, UK (E)	Sen, 1988
		5	1486	Host density × %	*Rhizophidium planktonicum*	*Asterionella Formosa*	Lake District, UK (OM)	Canter and Lund, 1953
		6	110	Host density × %	*Rhizophidium flagilariae, Chytridium versatile*	*Fragilaria crotonensis*	Lake District, UK (OM)	Canter and Lund, 1953
		0.04	1	Host density × %	*Rhizophydium couchii*	*Staurastrum* spp.	Lake District, UK (OM)	Canter and Lund, 1969
			1500	Host density × %	*Zygorhizidium* sp.	*Stephanodiscus parvus*	Lake Schohsee, Germany (M)	Holfeld, 1998
			170	Host density × %	Unknown monocentric chytrid	*Chrysamoeba radians*		
			660	Host density × %	*Rhizophydium planktonicum, R. tetragenum, Zygorhizidium planktonicum*	*Asterionella formosa*		
			40	Host density × %	*Zygorhizidium* sp.	*Fragilaria crotonensis*		
			9	Host density × %	*Zygorhizidium. planktonicum*	*Synedra acus*		
			30	Host density × %	*Haparopera piriformis*	*Ankyra judayi*		
			10	Host density × %	*Zygorhizidium parallelosede*	*Elakatothrix genevensis*		

*Minimum densities during the presence of chytrids were shown if available. The density of sporangia was estimated from the prevalence of infection and host cell density data from the literature (shown as “Host density × %”)*.

*CFW, calcofluor white; OM, oligo-mesotrophic; M, mesotrophic; E, eutrophic*.

* *Uncertain identification based on morphology or phylogeny*.

a *Asterionella, Fragilaria, Synedra, Staurastrum, Oocystis*.

b *Asterionella, Synedra, Staurastrum, Cyclotella, Fragilaria, Ankira, Melosira, Starodesmus, Chodatella, Ankystrodesms, Cylindrospermum, Oocystis*.

c *Asterionella, Synedra, Staurastrum, Cyclotella, Fragilaria, Ankira, Melosira, Oscillatoria, Microcystis, Fragilaria, Gomphosphaeria, Anabaena*.

Compared to quantifying the abundance of zoospores, sporangia are much easier to count because they are attached to host phytoplankton cells. Using prevalence of infection and host cell density data from the literature, we determined the density of sporangia to be 10^1^–10^8^ spores L^−1^ in field surveys (Table 1). The result indicated that direct counts of zoospores in the field may underestimate the real densities. By using the zoospore per sporangia conversion factors determined by previous studies (13–28 zoospores per sporangium, Sen, 1988; 4–25 zoospores per sporangium, Bruning, 1991), or the zoospore per sporangium biovolume conversion (0.166 per μm^3^ empty sporangium volume, Bruning, 1991), we can roughly estimate that zoospore abundance could actually reach more than 10^9^ zoospores L^−1^in the field. It should be noted that lowest abundance of zoospore can be 10 spores L^−1^, or even zero. This indicates that the potential importance of mycoloop may vary with seasons and lakes.

Molecular studies have also revealed that chytrid zoospore may often be miscounted as small heterotrophic nano-flagellates (HNF), due to similar forms and sizes (Sime-Ngando et al., 2011). A significant portion of small eukaryotes (0.6–5 μm) was recently found to be chytrid zoospores in freshwater lakes (11–23%, Lefèvre et al., 2007; 30% Lepère et al., 2008). In addition, CARD-FISH identified 5–60% of unknown flagellates as chytrids zoospores (Jobard et al., 2010). In aquatic ecosystems, most small heterotrophic eukaryotes (<5 μm) are considered to play a role in microbial food webs by acting as predators of bacteria and bacterium-sized phytoplankton (Sherr and Sherr, 1983). In contrast, chytrids consume phytoplankton directly as parasites, and they do not eat bacteria. These findings require that we should revise our understanding of microbial food webs.

Zoospores may become particularly important to *Daphnia* when large inedible phytoplankton species, such as the diatom *Asterionella*, dominate the phytoplankton community. Large phytoplankton species are quite resistant to grazing by zooplankton such as *Daphnia* (Knisely and Geller, 1986; Kagami et al., 2002). However, if large inedible phytoplankton species are infected by chytrids, then nutrients within host cells are consumed by chytrids and can be grazed by *Daphnia*. This new pathway has been dubbed the “mycoloop” since nutrients from large inedible algae are transferred to zooplankton via the zoospores of parasitic chytrids (Kagami et al., 2007a).

Trophic transfer efficiency from host algae to chytrids is an essential parameter to examine the importance of mycoloop in the field. The transfer efficiencies of carbon, nitrogen, and phosphorus (CNP) from host *Asterionella* populations to free-swimming zoospores were estimated to be 6–9% in the laboratory experiment, when the prevalence of infection was about 60% (Kagami et al., 2007b). Those efficiencies were population based, and may become even higher if the prevalence of infection may exceed 90%. While, a single zoospore can use the host tissues quite efficiently because chytrid can directly consume host nutrients by entering through a germ tube (Van Donk and Ringelberg, 1983). CNP concentrations in a single zoospore (10.7 pg C, 0.6 pg N, 2.4 pg P per zoospore, Kagami et al., 2007b) are comparable to 20% of those in single host cell, indicating just five zoospores may be enough to exploit all algal tissues. 20% must be overestimated, if we consider the range of zoospores per sporangia (4–25 zoospores per sporangium, Bruning, 1991). We need to measure the CNP concentrations of zoospores and host cells, and number of zoospores per sporangium accurately with different species and conditions. From these estimates, we can examine how important chytrid zoospores may be as a food source for zooplankton in the field, in comparison to other possible food sources. In addition, such estimates are also crucial for modeling approaches, to predict the roles of chytrids in altering the network structure and stability (Niquil et al., 2011), and in determining the zooplankton production (Miki et al., 2011).

The “mycoloop” may occasionally play an important role in shaping aquatic systems, by altering the material flow (Figure 1). Traditionally, large inedible phytoplankton species are believed to be lost by sinking from the euphotic zone (Malone, 1981). However, if large phytoplankton are parasitized, then nutrients within host cells are instead consumed by chytrids, and can, in turn, be grazed by *Daphnia* through the mycoloop (Kagami et al., 2007a). In addition, large inedible colonies of phytoplankton may be fragmented into smaller pieces due to chytrid infections, making them more edible to zooplankton (Figure 1, Sime-Ngando, 2012). The trophic transfer efficiency from large phytoplankton to *Daphnia* would not change largely even if the heavily infected colonies are fragmented and grazed (i.e., after most of the host cells are consumed by chytrid). It would change, however, if the lightly infected host colonies are fragmented and grazed (i.e., before most of the host cells are consumed by chytrid) (Sime-Ngando, 2012). In this way, nutrients in host phytoplankton cells are partly incorporated into the food web in the euphotic zone, instead of sinking.

On the other hand, some of the infected host colonies may remain inedible for *Daphnia*, or even become less edible due to the aggregation of cells (Kagami et al., 2005, Figure 1) and may sink faster than single colonies. In addition, frustules of previously infected cells may sink faster than living cells (Kagami et al., 2006). In this way, sinking of frustules and aggregates of host cells may actually be facilitated by chytrid infections.

## Predation on chytrids may suppress outbreaks of chytrids

Predation on the free-living stages of parasites may result in reduced disease risk for hosts (Packer et al., 2003). Indeed, the presence of *Daphnia* can decrease chytrid infection intensity on phytoplankton (Kagami et al., 2004). Recent studies also revealed that *Daphnia* grazing on free-swimming zoospores of *Batrachochytrium dendrobatidis* can decrease the disease chytridiomycosis of amphibians (Hamilton et al., 2012; Searle et al., 2013).

## Phytoplankton—chytrid—zooplankton interactions

Although a short-term experiment demonstrated that the direct trophic link from chytrid fungus to zooplankton (F-Z link) increased zooplankton growth (Kagami et al., 2007b), the effects of the F-Z link on food web dynamics is not easily predictable.

Considering that chytrid infections are common in large inedible phytoplankton species (Sommer, 1987; Kagami et al., 2007a), fungal parasitism may indirectly increase the abundance of small edible phytoplankton by altering resource competition. This may in turn enhance zooplankton production through grazing pathways via an “indirect mutualism” (Levine, 1976; Vandermeer, 1980). If the F-Z link then decreases the abundance of fungal zoospores (or fungal parasitism), it weakens the indirect mutualism between fungi and zooplankton, and will then decrease material transfer from small phytoplankton to zooplankton (indirect effect).

Therefore, the F-Z link may enhance zooplankton production through the mycoloop (direct effects), while it may also decrease zooplankton production by weakening indirect mutualism (indirect effect). By using a simple food web model, we successfully evaluated the effects of parasitic chytrids (fungal parasitism) and the F-Z link (both direct and indirect effects) on food web dynamics (Miki et al., 2011). In summary, presence of the F-Z link caused unexpected indirect effects in the food web, and was an important determinant for the stability of the system (see the following section for more detail). The model indicated that the high growth efficiency and high nutritional quality of fungi were crucial for the F-Z link to increase zooplankton production. The model also indicated that the contribution of the mycoloop (material transfer via the F-Z link) to zooplankton production increased with nutrient availability and depended on the system stability. This implies that neglecting the dynamical aspect of the system will lead to inaccurate estimates of material and energy fluxes. In the following section, we will review the theoretical approaches in detail to evaluate the roles of parasitic fungi in aquatic food webs.

## Multifaceted impacts of fungus-zooplankton interactions on food web dynamics: lessons from dynamical models

There are two modeling approaches for describing the structure, dynamics, and fluxes of material and energy in food webs and ecosystems: steady state models and dynamical models. The steady state model (or linear model) is a powerful tool in ecosystem sciences to quantitatively estimate material fluxes with limited observations (Vezina, 1989). For example, it has been used to estimate the impacts on carbon fluxes in aquatic ecosystems, of the microbial loop (e.g., Anderson and Ducklow, 2001; Anderson and Tang, 2010), bacteriophage (Fuhrman, 1999; Motegi et al., 2009), and the food web structure (Niquil et al., 2006). Recent studies also quantified the impact of chytrid fungi in lake carbon fluxes using this modeling approach with inverse estimates of fluxes (Grami et al., 2011; Niquil et al., 2011). On the other hand, the dynamical model, which often requires a larger number of parameters and more specific mathematical formulations for inter-compartment interactions (e.g., trophic and competitive interactions), can provide information about both the steady state structure and non-steady state dynamics of the food web. Here, we would like to highlight the three major impacts of the F-Z link on the food web; (1) effect on food web structure and zooplankton production, (2) influence on system stability, and (3) contribution to material fluxes (mycoloop), elucidated by dynamical food web models (Miki et al., 2011; Gerla et al., 2013). Since conclusions are often different between steady state models and dynamic models, we will compare these two modeling approaches.

**Figure 2:**
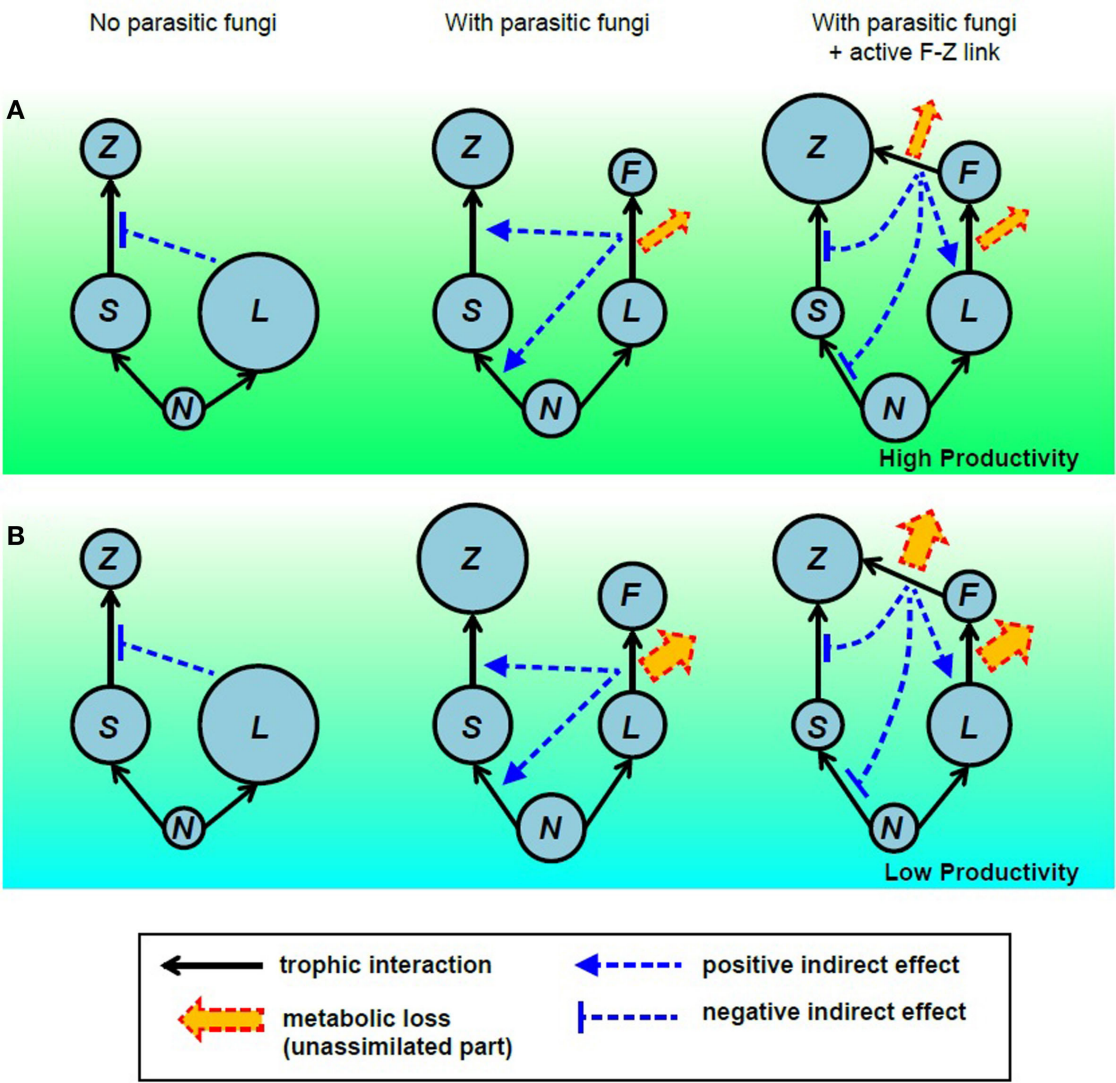
**Indirect effects of the F-Z link and their feedback on zooplankton**. The net effects of the fungus–zooplankton link on zooplankton biomass production depends on three conditions: the growth efficiency of fungi on the host, the growth efficiency of zooplankton consuming fungi, and the nutrient supply in the system. ***N***, inorganic nutrient; ***L***, large phytoplankton; ***S***, small phytoplankton; ***F***, chytrid fungi; and ***Z***, zooplankton. **(A)** When these growth efficiencies are high, in other words, when the metabolic loss of fungi or metabolic loss of zooplankton is low, or the system productivity is high, the F-Z link increases zooplankton biomass production, compared to the system with fungal parasitism only. **(B)** When metabolic losses are high or the system productivity is low, the F-Z link decreases zooplankton biomass production compared to the system with fungal parasitism only. More quantitative results are shown in Miki et al. (2011).

**Figure 3:**
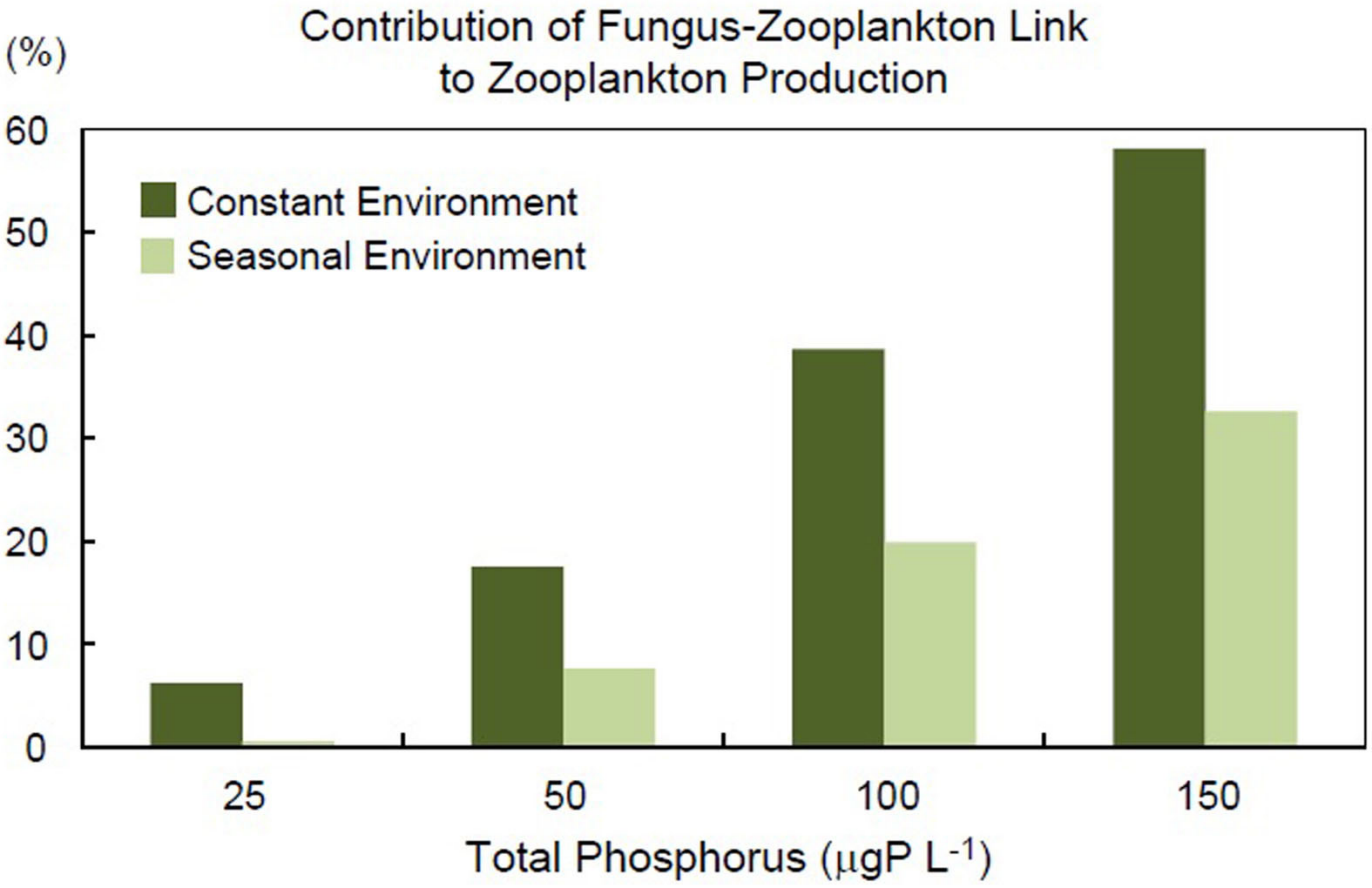
**Comparison of the contribution of the mycoloop pathway to zooplankton production under constant and seasonal environments**. The relative percent contribution of the mycoloop pathway (large phytoplankton → parasitic fungi → zooplankton) to total zooplankton biomass production under constant and seasonal environmental conditions was calculated as the ratio of the F-Z link nutrient flux to zooplankton compared to the total nutrient flux to zooplankton (nutrient flux from the F-Z link plus nutrient flux from small phytoplankton) (see also Figure 2). The ratios of the contribution of the mycoloop under constant environmental conditions compared to that under seasonal environmental conditions was calculated for concentrations of total phosphorus (TP) = 25.0, 50.0, 100, 150 μgP L^−1^ to be 10.3, 2.30, 1.94, 1.78. TP in the model ecosystem was calculated by the average phosphorus supply *I*_0_ divided by the turnover rate of the system (0.05/day). The daily fluctuation in the phosphorus supply *I*(*t*) is given by *I*(*t*) = *I*_0_ [1.0 + 0.5 sin(2π*t*/365)] for the seasonal environment; the maximum deviation from average is ±50%. Modified from Miki et al. (2011).

1. *Effects of the F-Z link on food web structure and zooplankton production:* The dynamical model can predict the unexpected consequences of nonlinear effects of adding or removing a specific trophic linkage or a specific player in the structure of the food web (Pimm, 1991). In our case, we added/removed two trophic linkages; fungal parasitism and the F-Z link (Miki et al., 2011) for a detailed comparison among three scenarios: the system without parasitic fungi, the system with fungal parasitism but without the F-Z link, and the system with both fungal parasitism and the F-Z link. The dynamical model predicts that the F-Z link indirectly lowers the abundance of small phytoplankton, altering the food web structure (Miki et al., 2011). This prediction agrees with the predicted decline in picophytoplankton production in Lake Pavin steady state model (Grami et al., 2011). Similarly, both models predict the positive impact of fungal parasitism and F-Z link on zooplankton production and biomass. This implies robust positive impacts of fungi on trophic transfer to higher trophic levels. Some scenarios in the dynamical model have not been explored in the framework of the steady state model. The dynamical model predicted that the F-Z link can unexpectedly reduce the production and biomass of zooplankton compared to a system with fungal parasitism but without an active F-Z link (Miki et al., 2011). This is unexpected considering the apparent (direct) benefit of the F-Z link to zooplankton. This occurs through an indirect effect; the F-Z link increases the host population (large inedible phytoplankton) via a top-down cascade, which in turn decreases the population of non-host, small edible phytoplankton through intensified resource competition (Figure 2). In particular, when the growth efficiency of parasitic fungi on host tissues and the nutrient quality of zoospores for zooplankton are not large enough (i.e., the metabolic loss through these trophic interactions is large) or productivity (nutrient availability) in the system is low, then indirect negative effects are greater than positive direct benefits and the F-Z link then causes a reduction in the zooplankton biomass and production (compare A vs. B in Figure 2). In order to better quantify the role of fungal parasitism and the F-Z link separately, three scenarios (the system without fungi, the system with fungal parasitism but without trophic transfer from fungi to zooplankton, and the system with both fungal parasitism and F-Z link) should be compared even with the steady state model approach.
2. *Influence of the F-Z link on system stability:* Although theoretical metrics for steady-state ecosystem structure can predict the complexity of the network and imply the stability of the system (Morris et al., 2005; Ulanowicz et al., 2009; Grami et al., 2011; Niquil et al., 2011), consequences of nonlinearity in trophic interactions on system stability can be evaluated more directly in a dynamical model. When the trophic interaction between host phytoplankton and parasitic fungi is parameterized by a prey–predator type model with non-linear functional response (e.g., Holling type II functional response) (Miki et al., 2011) or more explicitly parameterized by a host-parasite type model with SIV formulation (susceptible host, infected host, and free-living vector; Gerla et al., 2013), food web dynamics are predicted to be less stable than a model with a simple Lotka-Volterra type prey–predator functional response (Miki et al., 2011). In addition, although the network analysis implied a stabilization of the system by fungi (Grami et al., 2011; Niquil et al., 2011), the dynamical model clearly demonstrated that the presence of the F-Z link (Figure S1, Miki et al., 2011) or the presence of a host–fungus interaction itself (Figure 3, Gerla et al., 2013) can destabilize the system, especially in eutrophic conditions. The dependency of fungal zoospore production rate on host physiology and activity (e.g., nutrient uptake rate) is also proposed as the destabilizing factor (Gerla et al., 2013).
3. *Contribution of the mycoloop to material fluxes:* the above consideration on system stability implies that it may be risky to assume that the roles of parasitic fungi in material fluxes estimated by steady state model equates to their role under non-steady state conditions. A non-steady state dynamic of the food web may be caused internally by nonlinear trophic interactions (as mentioned above) or externally forced by environmental fluctuations. A steady-state model is able to provide a snapshot estimate of fluxes in an ecosystem even under non-steady-state conditions if the instantaneous mass accumulation rate in each ecosystem component is not too large (quasi-steady state assumption). However, the steady state assumption tends to significantly overestimate (a factor of 2–10) the annual averaged contribution of the F-Z link under seasonal fluctuations (Miki et al., 2011; Figure 3). More specifically, the predicted relative contribution of fungi to zooplankton production from the dynamical model under a stable environment is 38.6% when the growth efficiency of fungi on host phytoplankton is assumed to be 75% and the total phosphorus is 100 μgPL^−1^ (Figure 3). This prediction is comparable to the estimate of the contribution of fungal zoospores in the total diet of microzooplankton in ologimesotrophic Lake Pavin (38%) (Grami et al., 2011). However, an introduction of seasonality into the dynamical model lowers the contribution of fungi to 19.9%. Such an overestimate is a general feature in nonlinear systems. When the trophic flux (*F*) is proportional to the abundance of resources (*R*) and consumers (*C*): *F* = *aRC* where *a* is the consumption coefficient, then the average flux (*F*(*t*)) is not equivalent to the product of the averages *R*(*t*) and *C*(*t*). Instead, we have *F(t)* = *a*[*R(t)* · *C(t)* + Cov (*R*(*t*),*C*(*t*))], implying that neglecting the impacts of asynchronous population dynamics of resources and consumers (Cov(*R*,*C*) < 0) is the source of the overestimation with a steady-state assumption.

Combination of steady state models and dynamical models are a promising approach to greatly improve our understanding of the roles of parasitic fungi.

## Future perspectives

### Molecular tools

Recent advances in molecular methods enable us to investigate the species composition of microorganisms. Indeed, several methods, such as PCR-DGGE, clone libraries, FISH, and qPCR have been applied to describe the species composition and biomass of certain species of parasitic chytrids (Jobard et al., 2010; Lefèvre et al., 2010; Kagami et al., 2012; Marano et al., 2012, Maier et al., under revision). In addition, next generation sequencing will be beneficial for the analysis of fungal community structures. However, since the DNA database of aquatic fungi is scarce, especially for the parasitic fungi (chytrids), it is difficult to determine species composition and function (e.g., parasitic or saprotrophic) by analyzing environmental DNA alone. In addition, choosing the right primer sets are essentially important when examining the species composition by molecular tools (Wurzbacher et al., 2010, Ishii et al., in review). Therefore, prior to applying advanced molecular tools, we first need to build a robust database, especially for the parasitic chytrids. Culturing, single cell PCR methods, and whole genome sequencing will aid in having a better understanding of the community structure and function of parasitic fungi wide-ranging ecosystems.

### Other possible mycoloops

**Figure 4:**
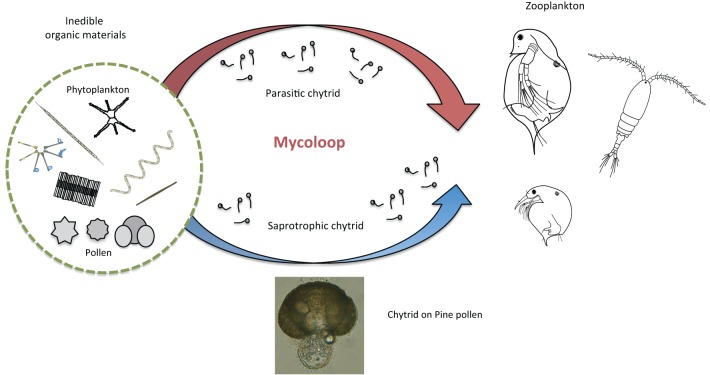
**Other possible mycoloops in freshwater and marine environments**. Saprotrophic chytrids may play important roles in aquatic food webs, by decomposing inedible organic material such as pollens. Zoospores released from pollen may be consumed by zooplankton, functioning as another “mycoloop.” In addition to chytrids, other zoosporic fungi or fungal-like protists, such as Cryptomycota and Labyrinthulomycota, can infect phytoplankton or consume large inedible organic material, which may be grazed by zooplankton in freshwater and marine environments.

In addition to parasitic chytrids, saprotrophic chytrids may also play important roles in aquatic food webs. For instance, pollen deposition into lakes may not be utilized directly by zooplankton, but can be decomposed/consumed by saprotrophic chytrids (Masclaux et al., 2011, 2013). Grazing of zoospores released from pollen may then function as another “mycoloop” (Figure 4).

Recently discovered fungi, the Cryptomycota, exhibit a similar life cycle to chytrids including a free-swimming stage, and are also known to infect phytoplankton (Jones et al., 2011). In marine environments, some Chytridiomycota or zoosporic fungal-like protists such as Labyrinthulomycota are also known to infect marine phytoplankton (Raghukumar, 2002; Gleason et al., 2011), and may play important roles in marine food web dynamics (Raghukumar, 2002). These results indicate the existence of other possible mycoloops in freshwater and marine ecosystems via the route of free-swimming zoospores of newly discovered Chytridiomycota, Cryptomycota, or Labyrinthulomycota.

### Conflict of interest statement

The authors declare that the research was conducted in the absence of any commercial or financial relationships that could be construed as a potential conflict of interest.
